# The Influence of Heat Treatment Process on the Residual Ferrite in 304L Austenitic Stainless Steel Continuous Casting Slab

**DOI:** 10.3390/ma18163724

**Published:** 2025-08-08

**Authors:** Zhixuan Xue, Kun Yang, Yafeng Li, Chaochao Pei, Dongzhi Hou, Qi Zhao, Yang Wang, Lei Chen, Chao Chen, Wangzhong Mu

**Affiliations:** 1 College of Materials Science and Engineering, Taiyuan University of Technology, Taiyuan 030024, China; 2024510282@link.tyut.edu.cn (Z.X.); 2024520357@link.tyut.edu.cn (C.P.); 2023000825@link.tyut.edu.cn (D.H.); zhaoqi0825@link.tyut.edu.cn (Q.Z.); wangyang0291@link.tyut.edu.cn (Y.W.); 2 College of Mechanical Engineering, Taiyuan University of Technology, Taiyuan 030024, China; yangkun@tyut.edu.cn (K.Y.); chenlei839@163.com (L.C.); 3 Xinzhou Comprehensive Inspection and Testing Center, Xinzhou 034000, China; 18003545064@163.com; 4 Department of Materials Science and Engineering, KTH Royal Institute of Technology, SE-100 44 Stockholm, Sweden

**Keywords:** 304L austenitic stainless steel, continuous casting slab, residual ferrite, thermodynamic calculations, heat treatment experiment

## Abstract

This study investigates the distribution characteristics of residual ferrite in 304L austenitic stainless steel continuous casting slab and the impact of heat treatment processes on its content. Through optical microscopy (OM), thermodynamic calculation software (Thermo–Calc) and heat treatment experiments, it is found that the residual ferrite content along the thickness direction at the width center of the slab exhibits an “M”-shaped distribution—lowest at the edges (approximately 3%) and highest near the center (approximately 13%). Within the triangular zone of the slab, the residual ferrite content varies between 1.8% and 12.2%, with its average along the thickness direction also showing an “M”-shaped distribution; along the width direction, the average residual ferrite content is lower at the edge positions, while within the internal triangular zone, it ranges between 8% and 10%. The ferrite morphology changes significantly across solidification zones: elongated in the surface fine-grain zone, lath-like and skeletal in the columnar grain zone and network-like in the central equiaxed grain zone. Thermodynamic calculations indicate that the solidification mode of the 304L continuous casting slab follows the FA mode. Heat treatment experiments conducted across the entire slab thickness demonstrate effective reduction in residual ferrite content; the optimal reduction is achieved at 1250 °C with a 48 min hold followed by air cooling while preserving the original “M”-shaped distribution characteristic after treatment. Increasing the heat treatment temperature, prolonging the holding time and reducing the cooling rate all contribute to reducing residual ferrite content.

## 1. Introduction

As a typical grade of high-alloy steel, stainless steel exhibits excellent corrosion resistance and heat resistance, making it widely applicable in industrial fields and daily life [1,2,3,4,5,6]. Due to its outstanding corrosion resistance and mechanical properties, 304L austenitic stainless steel is commonly used in engineering sectors, such as petrochemicals, rail vehicles and aerospace [7,8,9]. The microstructure of austenitic stainless steel produced via continuous casting typically features a ferrite–austenite duplex structure. The morphology and content of ferrite in the surface solidified structure of the cast slab can induce edge cracking during hot rolling [10,11,12,13]. Statistical evidence indicates that the presence of excessive ferrite (usually above 10%) adversely affects the corrosion resistance of cast slab [14,15,16]. When the ferrite content in 304L austenitic stainless steel is low, it essentially becomes a fully austenitic phase. This not only provides corrosion resistance but also ensures non-magnetic properties. Consequently, it is widely used in fields requiring non-magnetic interference, such as nuclear power, medical devices and mobile phone casings. The presence of the ferrite phase limits the application of 304L austenitic stainless steel [17,18]. Therefore, controlling the ferrite content in the continuous casting slab of 304L austenitic stainless steel is crucial [19,20,21,22,23,24].

In the conventional continuous casting process, where cooling is achieved through surface spray water cooling, the heat flow within the slab cross-section transfers unidirectionally along the thickness direction. This results in non-uniform cooling rate distribution across the slab thickness, leading to variations in ferrite content along this direction. The inhomogeneous distribution of residual ferrite across different regions of the slab will confer anisotropic mechanical properties and the formation of corrosion-susceptible zones (where ferrite-enriched areas are prone to selective corrosion). Therefore, research on ferrite distribution in different slab regions is critically important [25,26,27,28,29]. Numerous researchers have investigated ferrite content along the thickness direction in austenitic stainless steels. As early as 1984, Lindenberg et al. [30] first reported the inhomogeneous distribution of ferrite in stainless steel cast slab. Wolf et al. [31] found irregular ferrite distribution in 304 stainless steel slab. In 1995, Kim et al. [32] observed that ferrite content in 304 austenitic stainless steel slab exhibited an “M”-shaped distribution along the thickness direction: approximately 4% at the surface, reaching a maximum of 9% at 95 mm from the surface and decreasing to 6% at the slab center. Moreira dos Santos et al. [33] studied the formation and evolution of δ-ferrite during the hot processing of an 80-ton industrial AISI 304 austenitic stainless steel slab. Their research confirmed the “M”-shaped distribution of ferrite content along the thickness direction. After two subsequent hot rolling processes and one solution heat treatment, the average δ-ferrite content decreased following each treatment. Huang et al. [34,35] studied the δ-ferrite distribution in a billet and continuous cast strip. They found an “M”-shaped distribution of ferrite content along the thickness direction of the billet and a “W”-shaped distribution of ferrite content in the strip.

In summary, extensive research has demonstrated that ferrite content in 304 austenitic stainless steel cast slab follows an “M”-shaped distribution along the thickness direction. This pattern primarily arises from variations in composition, cooling rates and solidification modes across the slab cross-section. However, existing studies have predominantly focused on ferrite distribution at the width center of the slab, with limited research on ferrite content distribution at other positions. Chen and Cheng [36] investigated ferrite distribution at different positions across the cross-section of Fe-Cr-Mn stainless steel cast slab. They also observed an “M”-shaped distribution of ferrite content along the thickness direction in 304 stainless steel slab. Only Spacarotella et al. [37] examined the distribution of ferrite along the thickness direction at various positions across the entire cross-section of a 304 stainless steel slab, similarly identifying the characteristic “M”-shaped pattern.

Currently, heat treatment methods can be employed to regulate the ferrite content in austenitic stainless steel cast slab. Most researchers have focused on the changes in ferrite content under different heat treatment regimes. Nhung et al. [38] investigated the influence of temperature on δ-ferrite content in austenitic stainless steel welds. Specimens were held at 400 °C, 600 °C and 900 °C for 10 h. The study revealed that δ-ferrite content gradually decreased with increasing temperature, dropping from 23.5% in the as-welded state to 22% after holding at 400 °C for 10 h and further reducing to 11% after holding at 900 °C for 10 h. Kim et al. [39] explored the dissolution kinetics of δ-ferrite in 304 stainless steel strip specimens by varying annealing times within the temperature range of 1050–1200 °C. They found that δ-ferrite could generally be eliminated through prolonged homogenization heat treatment within this temperature range. Fukumoto et al. [40] studied the dissolution behavior of δ-ferrite in SUS304 continuous cast slab within the heat treatment temperature range of 1100–1200 °C. Specimens were heated for 20–120 min followed by water cooling. The results indicated that the δ-ferrite dissolution rate increased with rising temperature within the 1100–1200 °C range. Microstructural analysis showed a decreasing trend in δ-ferrite quantity, with its morphology transforming from vermicular to rod-shaped and spherical. Aghebatkheiri et al. [41] investigated the dissolution kinetics of δ-ferrite during homogenization of AISI 304 austenitic stainless steel. The study covered a temperature range of 1050–1250 °C and time spans of 1–12 h, with hourly sampling. The results demonstrated that δ-ferrite content decreased with increasing annealing temperature and time. Zargar et al. [42] used Thermo-Calc software to predict the “M”-shaped δ-ferrite content distribution along the thickness direction of continuously cast 304 austenitic stainless steel slab. A comparison between observations and calculations indicated that under rapid cooling conditions, reducing the cooling rate during solid-state transformation at the surface effectively decreased residual δ-ferrite. Li et al. [43] studied the hot deformation behavior of 301L stainless steel at 1000–1250 °C and strain rates of 0.1–50 s^−1^. Diffusion annealing reduced δ-ferrite content from 12% to 0.67%, with δ-ferrite dissolution controlled by chromium and nickel diffusion, particularly evident at the γ/δ phase interfaces. The optimal diffusion annealing parameters were determined as 1300 °C for 10 min. As early as 1990, Wang et al. [44] also investigated changes in ferrite content in 316L stainless steel cast slab after heat treatment. They found that ferrite content decreased by nearly half after treatment while still maintaining the characteristic “M”-shaped distribution. However, most existing research has focused on laboratory-scale specimen heat treatment experiments. Subsequent studies on ferrite distribution within actual cast slab after industrial heat treatment have been scarce.

This study focuses on 304L austenitic stainless steel slab, investigating the morphological variations in ferrite along the slab thickness direction. The ferrite distribution in both the triangular zone of the slab and along the thickness direction at the width center of the cast slab is examined. Simultaneously, Thermo-Calc thermodynamic calculation software is employed to simulate the solidification process of the 304L cast slab. Based on the thermodynamic calculation results, various heat treatment processes are designed. Heat treatment experiments are then conducted across the entire slab thickness to investigate the reduction in ferrite content and the changes in ferrite distribution under different heat treatment regimes.

## 2. Materials and Methods

**Figure 1:**
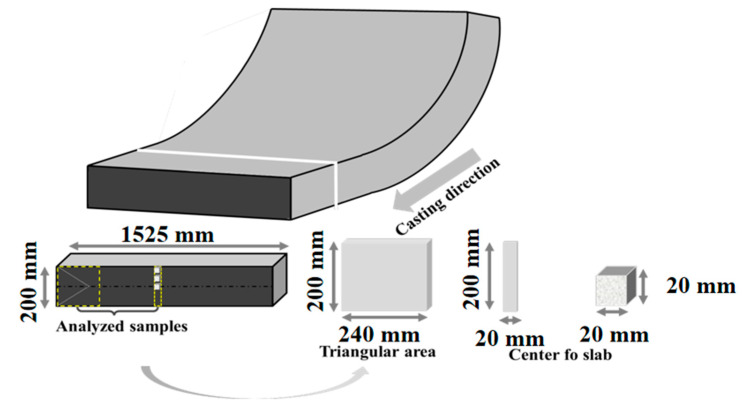
Schematic diagram of sampling of the continuous casting slab.

**Figure 2:**
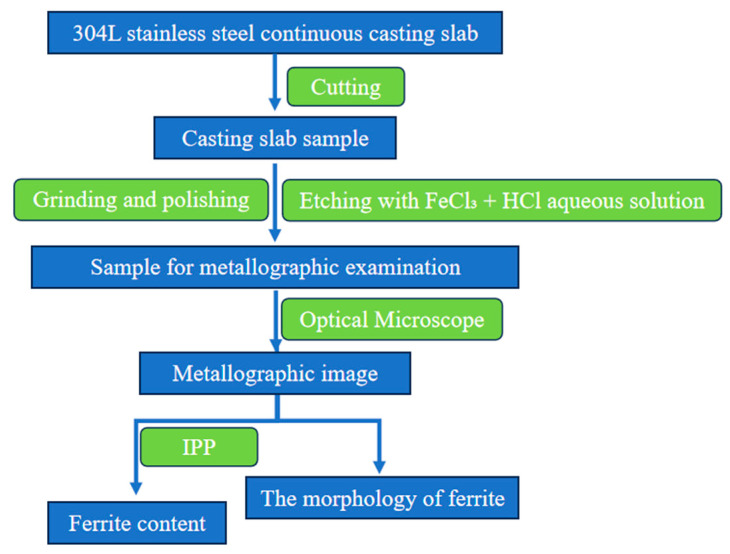
Metallographic process flow diagram.

A batch of 304L continuous casting slab produced by a certain factory was selected, with a cross-section size of 200 mm × 1525 mm. Its composition is shown in Table 1. Samples were taken from both the triangular zone and the width center position of the slab. The metallographic method [45,46,47] was used to mechanically grind each sample sequentially using 400–2000 grit diamond sandpaper. Subsequently, polishing was performed using SiC polishing paste. The samples were etched with an aqueous HCl + FeCl_3_ solution for approximately 2 min to reveal the ferrite structure. The ferrite content on the etched surface of each sample was determined using a ZEISS AX10 optical microscope (Precise Instrument Co., Ltd., Beijing, China.)at 100× magnification. To cover all areas of the sample surface, ferrite was observed and measured in at least 10 positions. The volume fraction of ferrite in the selected positions was measured using IPP (version: Image-Pro-Plus 7.0) software, and the average value was calculated. Additionally, samples were taken from different positions at the slab center to observe the ferrite morphology; the sampling schematic is shown in Figure 1. The process flow of the metallographic method is shown in Figure 2.

**Figure 3:**
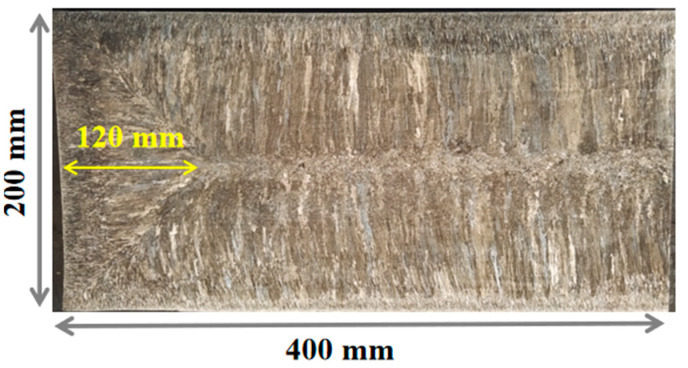
Macrostructure at the edge of 304L continuous casting slab.

To investigate the relationship between the solidification structure and the distribution of residual ferrite, the slab was subjected to macroscopic etching using an HCl + HNO_3_ aqueous solution. The etching results of the slab are shown in Figure 3.

To investigate the equilibrium solidification process of the 304L continuous casting slab, the phase evolution during solidification from 1500 °C to 500 °C was calculated using Thermo-Calc (version: Thermo-Calc 2025a) software based on the composition in Table 1. Building upon these thermodynamic calculations, 20 mm × 20 mm × 200 mm specimens were extracted from the center of the 304L slab. These specimens were heated in the BZ-4-13 muffle furnace (Kejing Material Technology Co., Ltd., Hefei, China) to four target temperatures (1000 °C, 1200 °C, 1250 °C and 1300 °C) and held for two sets of holding times (32 min and 48 min). After holding, rapid cooling was performed using either water cooling or oil cooling to obtain room-temperature microstructures. The specific experimental design is detailed in Table 2. Samples were taken from different positions of the heat-treated 304L slab to measure the residual ferrite content, following the same methodology described previously. Additionally, the through-thickness distribution of residual ferrite in the slab was measured under different heat treatment conditions.

## 3. Results

### 3.1. Equilibrium Solidification Process of 304L Slab

**Figure 4:**
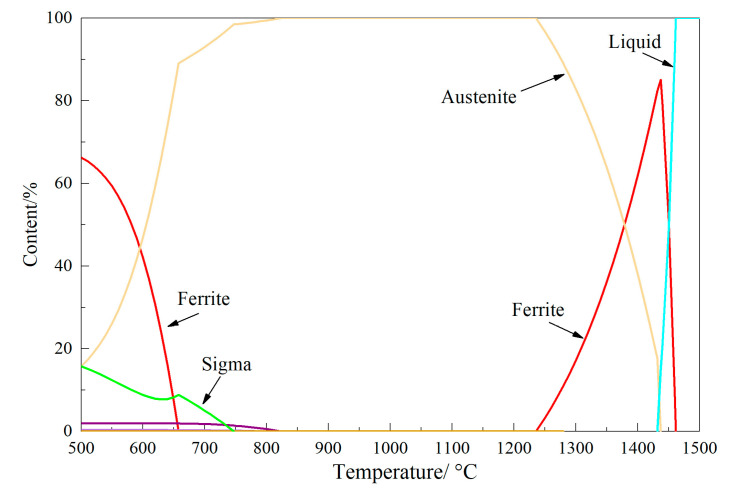
Equilibrium solidification process of the 304 stainless steel calculated by Thermo-Calc.

To investigate the evolution of residual ferrite during the heating and rolling process of continuous casting slab, Thermo-Calc software was employed to calculate the phase transformation behavior of 304L stainless steel during solidification from 1500 °C to 500 °C, as shown in Figure 4. The results indicate the following: At 1462 °C, δ-ferrite begins to form in the liquid phase. When the temperature decreases to 1437 °C, austenite starts to precipitate in the liquid phase, while δ-ferrite content reaches its peak value of 85.1%. With further cooling, δ-ferrite content gradually decreases as austenite content increases. At 1432 °C, the liquid phase completely disappears, with δ-ferrite content at 82.3%. When the temperature drops to 1236 °C, δ-ferrite vanishes, leaving austenite as the sole constituent in the microstructure. 1437–1462 °C: L + δ dual-phase region. 1432–1437 °C: L + δ + γ three-phase region. 1236–1432 °C: δ + γ dual-phase region. 823–1236 °C: γ single-phase region. The solidification mode of austenitic stainless steels refers to distinct solidification sequences, typically categorized as A, AF, FA or F patterns based on the precipitation order of ferrite or austenite. The AF and FA modes specifically denote solidification processes where ferrite or austenite, respectively, serves as the primary phase for nucleation and growth. As illustrated in Figure 4, the solidification sequence follows L→L + δ→L + δ + γ→δ + γ→γ→γ + α + precipitates. This progression indicates that the solidification mode conforms to the FA (Ferritic-Austenitic) pattern in stainless steels.

### 3.2. Morphology and Distribution of Residual Ferrite at the Mid-Width of Continuous Casting Slab

**Figure 5:**
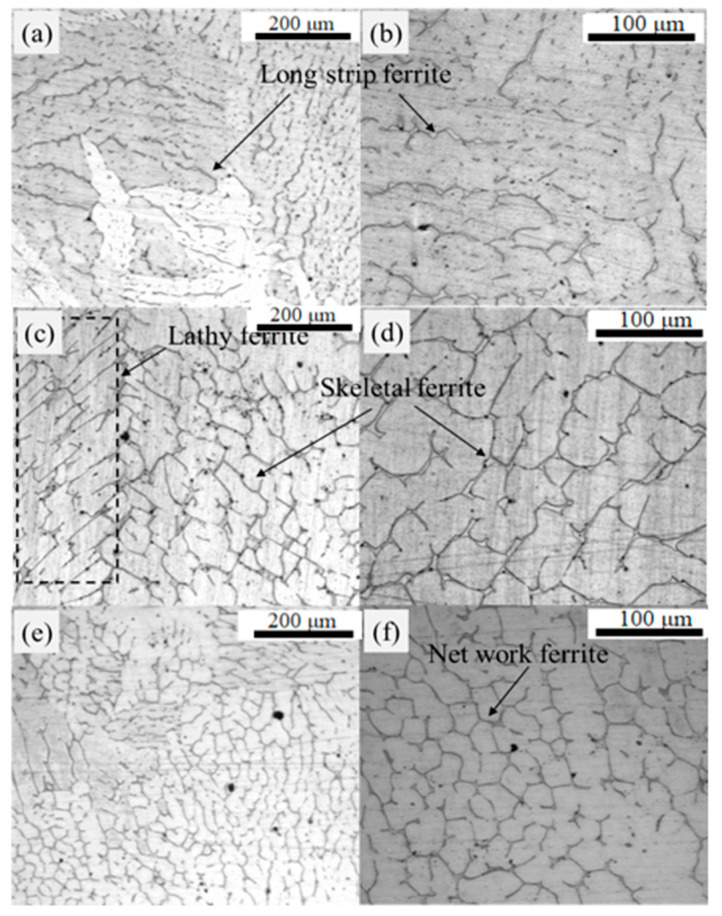
Ferrite morphology in different macrostructural regions of the slab: (**a**,**b**) Surface fine-grain zone; (**c**,**d**) Columnar crystal zone; (**e**,**f**) Central equiaxed crystal zone.

In the macrostructure shown in Figure 2, three primary regions are observed outside the triangular zone of the 304L slab: the surface fine-grain zone, columnar crystal zone and central equiaxed crystal zone. The morphology of ferrite along the thickness direction at the slab center is presented in Figure 5. Specifically, Figure 5a,c,e display ferrite morphology at 100× magnification in the surface fine-grain zone, columnar crystal zone and central equiaxed crystal zone, respectively. Figure 5b,d,f show corresponding ferrite morphology at 200× magnification.

The solidification structure of the continuous casting slab is predominantly characterized by columnar crystals, with relatively small proportions of surface fine-grain and central equiaxed crystal zones. The central solidification structure exhibits normal morphology without defects such as shrinkage porosity or cavities. As shown in Figure 5a,b, long strip ferrite predominates in the surface fine-grain zone. These long strip ferrite structures display parallel alignment, with some containing fine secondary dendrites. The columnar crystal zone (Figure 5c,d) reveals both lathy and skeletal ferrite morphologies. These lathy and skeletal structures are retained due to incomplete transformation from ferrite to austenite under FA solidification mode [48]. In contrast, the central equiaxed crystal zone (Figure 5e,f) features a network-like ferrite structure enveloping austenite phases.

**Figure 6:**
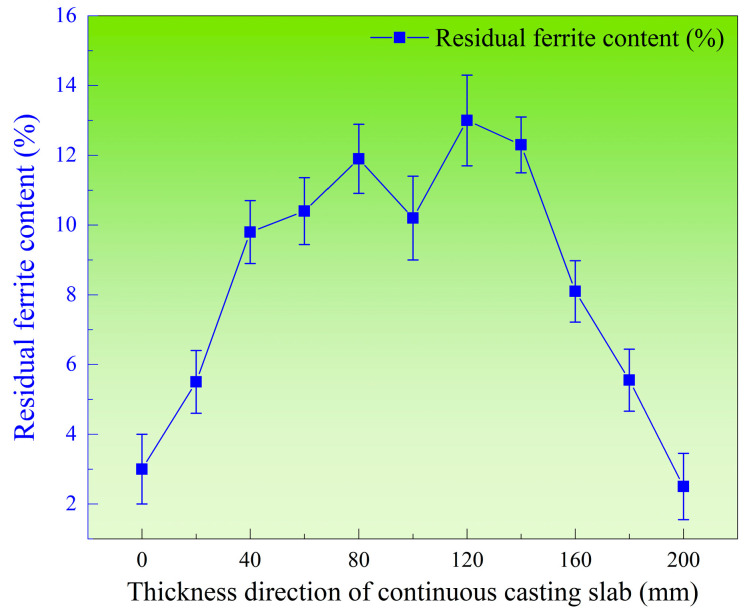
Distribution of residual ferrite in the thickness direction at the width center.

Further analysis of the ferrite content distribution along the thickness direction at the mid-width of the slab is presented in Figure 6. The ferrite distribution exhibits a distinct “M”-shaped profile at the slab center, with the lowest ferrite content of 3% observed near the slab edges and the highest content reaching 13% in the vicinity of the slab center. The ferrite content progressively increases from the slab surface to a depth of 80 mm but subsequently decreases toward the central region. Compared with the ferrite morphologies in Figure 5, significant variations are observed at the slab center. The formation of networked ferrite in the central equiaxed crystal zone may account for the reduced ferrite content in this region.

### 3.3. Distribution of Residual Ferrite in the Triangular Zone Region of Continuous Casting Slab

Table 3 presents the distribution of residual ferrite in the edge region of the 304L continuous casting slab. As shown in Table 3, the ferrite content exhibits significant variation within the slab. The minimum ferrite content of 1.8% occurs at the width edge, while the maximum value reaches 12.5% near the slab center. Residual ferrite content progressively increases along the through-thickness direction from the surface toward the center, followed by a moderate decrease at the central location.

**Figure 7:**
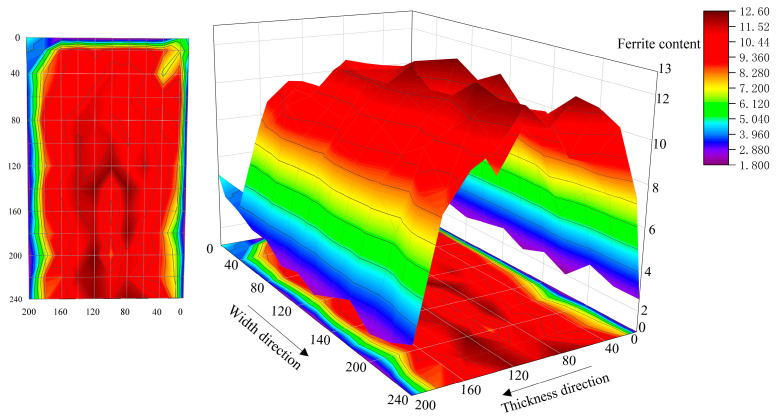
Three-dimensional distribution map of residual ferrite in 304L continuous casting slab.

The distribution of residual ferrite in the continuous casting slab was visualized through a three-dimensional representation, as shown in Figure 7. Analysis of Figure 7 reveals that residual ferrite content is relatively low near the slab edges and higher at the slab center. Within the triangular zone, ferrite content progressively increases from the width edge toward the center. A distinct ferrite content maximum occurs at the apex of the triangular zone, consistent with findings reported by Chen and Cheng [36] and Spacarotella et al. [37]. In regions outside the triangular zone, ferrite content gradually increases along the through-thickness direction from the slab edges toward the center. The distribution exhibits bilateral symmetry across the thickness direction. A ferrite content maximum appears near the thickness center. Ferrite content shows a moderate decrease precisely at the thickness center. Overall, the distribution displays a pronounced “M”-shaped profile.

**Figure 8:**
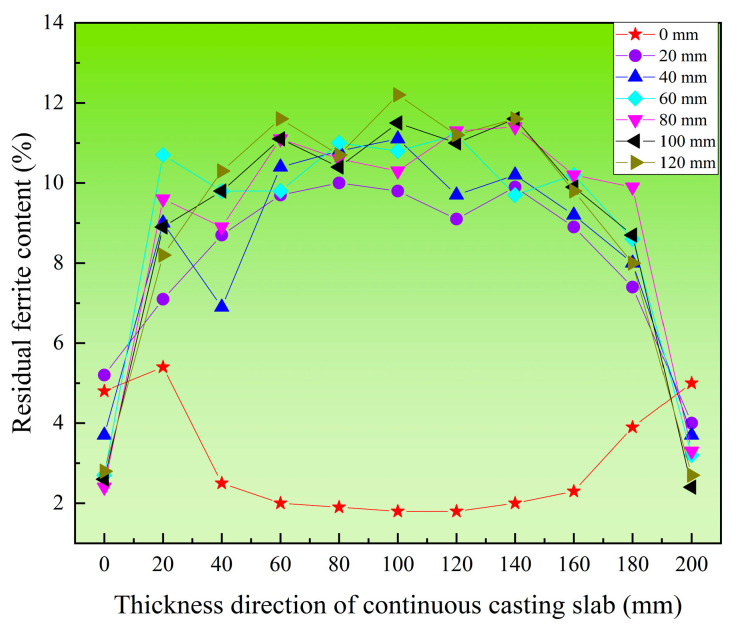
Distribution of residual ferrite along the thickness direction at different distances in the width direction of continuous casting slab (within the triangular area).

To further analyze the distribution patterns of ferrite along the thickness direction at different width positions, Figure 8 shows the residual ferrite distribution along the thickness direction within the 0–120 mm range (inside the triangular zone) along the slab width. In the edge region along the width direction, the ferrite content is lower, exhibiting a “V”-shaped distribution. This may be related to the irregular shrinkage profile at the slab width edge shown in Figure 3. This phenomenon occurs because the slab edge undergoes solidification shrinkage during the solidification process and does not fully contact the mold, resulting in non-uniform cooling rates. In areas with severe solidification shrinkage, the excessive cooling rate leads to lower ferrite content. From the 20 mm width position to the end of the triangular zone, the ferrite distribution along the entire thickness direction consistently shows an “A”-shaped pattern. The ferrite content is relatively low in the edge regions along the thickness direction, while it remains relatively consistent from the 20 mm thickness position to the center of the thickness. Within this range, the ferrite content is maintained between 8% and 12%.

**Figure 9:**
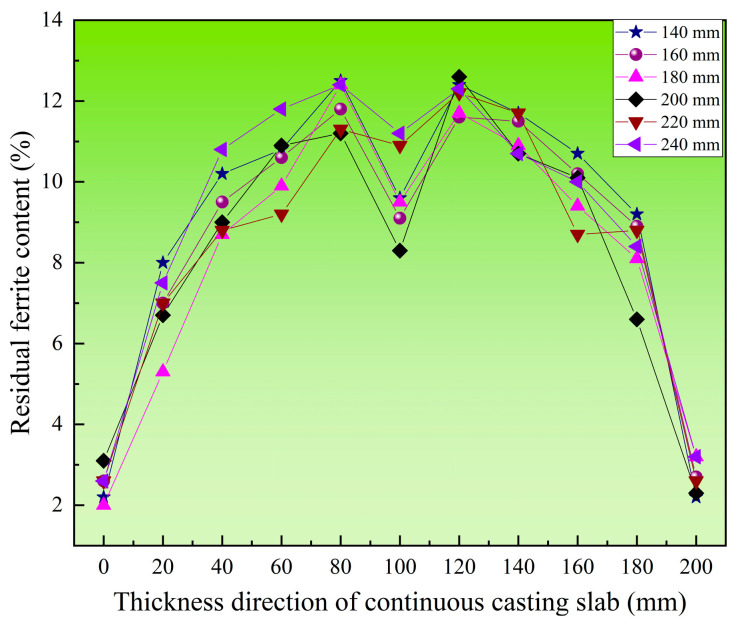
Distribution of residual ferrite along the thickness direction at different distances in the width direction of continuous casting slab (outside the triangular area).

Figure 9 shows the residual ferrite distribution along the thickness direction within the 140–240 mm range (outside the triangular zone) along the slab width. In the region outside the triangular zone of the slab, it can be observed that the residual ferrite content at different positions along the width direction clearly exhibits an “M”-shaped distribution. Moreover, the residual ferrite content shows minimal variation across these positions. At the center of the slab thickness, a distinct reduction in ferrite content is evident.

**Figure 10:**
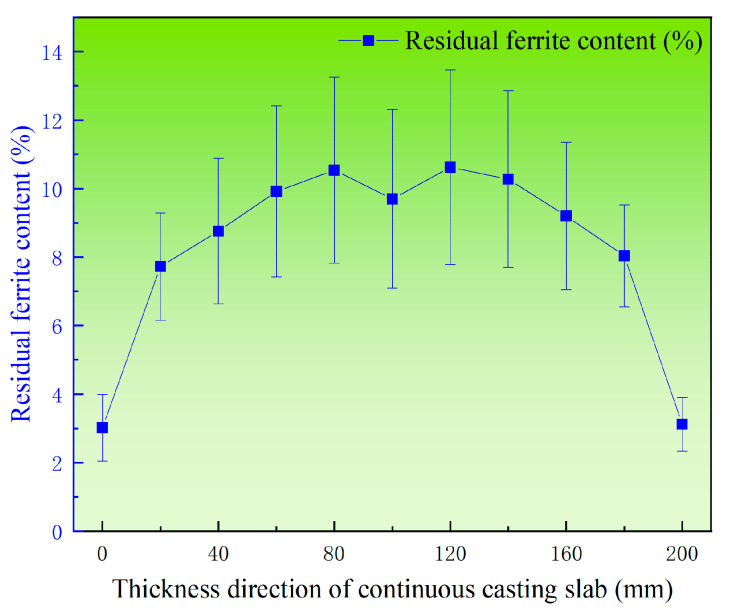
Distribution of average residual ferrite along the thickness direction of 304L continuous casting slab.

Additionally, the average values and standard deviation of residual ferrite along both the width and thickness directions were calculated separately. Figure 10 illustrates the distribution of average residual ferrite content along the thickness direction in the triangular zone of the continuous casting slab. It can be observed that along the slab thickness direction, the residual ferrite distribution exhibits an “M”-shaped pattern similar to that observed at the width center of the slab. The lowest average residual ferrite content occurs at the edge of the thickness direction, measuring 3.02%. From the edge to a position 80 mm from the edge, the average ferrite content gradually increases, peaking at 11.8%. Subsequently, toward the slab center, the average ferrite content decreases to 9.1%.

**Figure 11:**
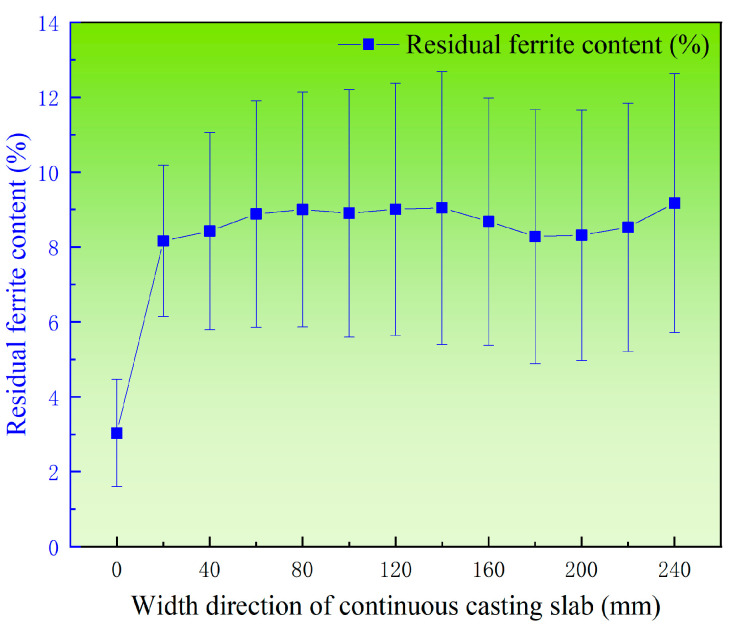
Distribution of average residual ferrite along the width direction of 304L continuous casting slab.

Figure 11 shows the distribution of average residual ferrite content along the width direction in the triangular zone of the continuous casting slab. It can be observed that along the slab width direction, the ferrite content remains relatively consistent within the slab interior, ranging between 8% and 10%, except at the edge, where the residual ferrite content is significantly lower at 3.04%.

### 3.4. Distribution of Residual Ferrite in the Triangular Zone Region of Continuous Casting Slab

**Figure 12:**
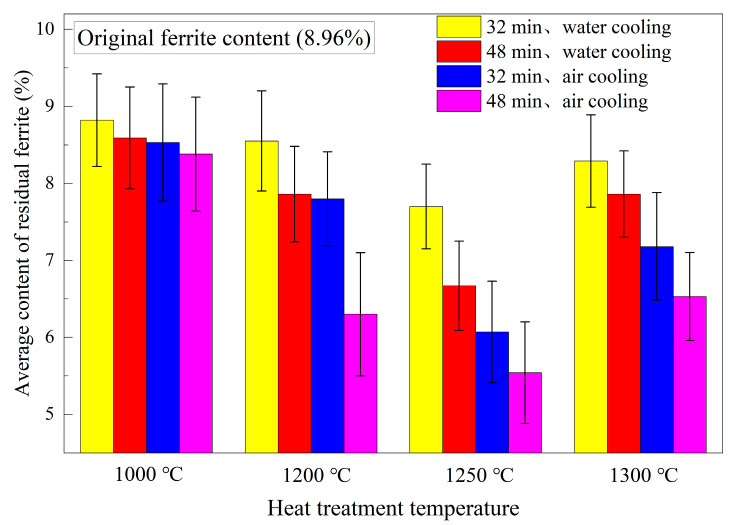
Average value of residual ferrite content after different heat treatment tests.

Additionally, eleven sampling points were selected across the entire thickness direction of the obtained continuous casting slab specimens for ferrite content measurement. The average ferrite content along the thickness direction was calculated from three repeated experimental measurements. Comparing the changes in average residual ferrite content before and after heat treatment, Figure 12 demonstrates that the residual ferrite in all heat-treated slabs decreased to varying degrees.

Figure 12 presents the average residual ferrite content for 16 sets of specimens after testing, with the original average ferrite content being 8.96%. The results reveal that under the same heat treatment temperature, specimens subjected to air cooling exhibit a more significant reduction in residual ferrite compared to water cooling. Under identical cooling conditions, extended holding times correspond to greater reductions in residual ferrite. Comparing the results across different temperatures, increasing the heat treatment temperature promotes the transformation of residual ferrite into austenite, thereby reducing ferrite content. Paradoxically, the reduction at 1300 °C was less pronounced than under 1250 °C holding conditions. The most substantial reduction occurred at 1250 °C, with the maximum decrease observed after 48 min of air cooling, reducing the ferrite content to 5.54% (a reduction of 3.42 percentage points). This phenomenon is explained by Thermo-Calc calculations (version: Thermo-Calc 2025a) (Figure 4) of phase evolution in 304L steel. The equilibrium microstructure at 1300 °C consists of high-temperature δ-ferrite and austenite, which is unconducive to residual ferrite transformation. δ-ferrite disappears at 1236 °C, and following further temperature reduction, it enters the fully austenitic phase region. Consequently, the most significant average ferrite reduction occurs following heat treatment at 1250 °C.

**Figure 13:**
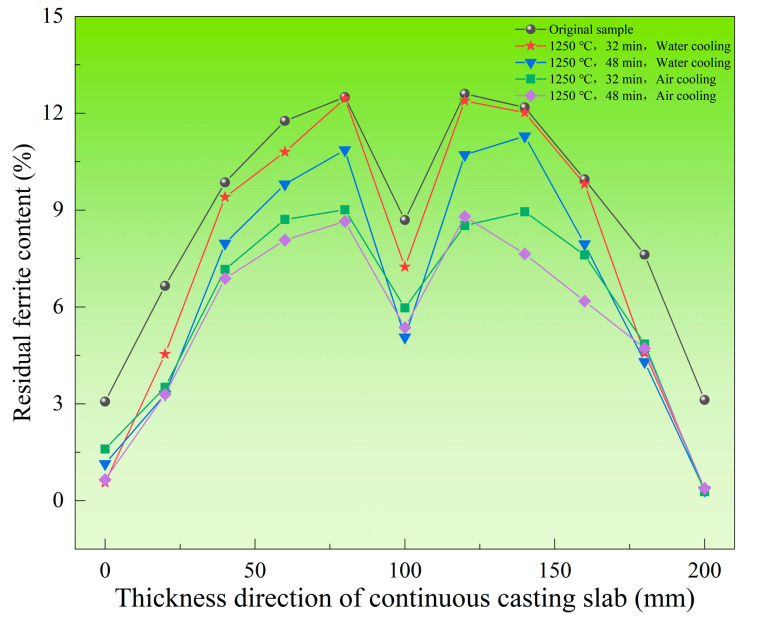
Comparison diagram of residual ferrite content between heat-treated sample and original sample of 304L continuous casting slab at 1250 °C.

To investigate the effect of heat treatment on the residual ferrite distribution in continuous casting slab, a comparative analysis was conducted between four sets of specimens heat-treated at 1250 °C and the original specimens, as illustrated in Figure 13. The results demonstrate that after heat treatment at 1250 °C, the residual ferrite distribution in the slab remains heterogeneous and retains the original “M”-shaped pattern observed in the as-cast slab. Under identical cooling methods, the ferrite content in the slab after 48 min holding is generally lower than that after 32 min holding. When holding times are equivalent, air-cooled slabs exhibit lower residual ferrite content than water-cooled slabs. Notably, however, within the chill layer at the slab edge, water cooling induces a more pronounced reduction in residual ferrite content.

### 3.5. Actual Precipitation Behavior of α-Ferrite

**Figure 14:**
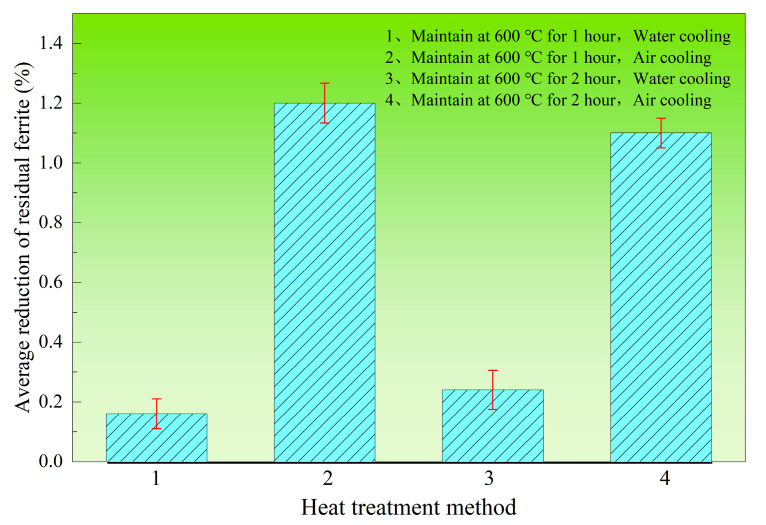
The changes in residual ferrite in 304L continuous casting slab were tested at 600 °C.

Thermo-Calc calculations in Figure 4 indicate that α-ferrite appears in the equilibrium phase structure when the temperature drops to approximately 600 °C. To verify whether α-ferrite increases during actual production processes, four specimen groups were subjected to prolonged holding at 600 °C followed by different cooling methods. The resulting changes in residual ferrite content (average reduction) are shown in Figure 14. The key findings are as follows. Contrary to thermodynamic predictions, residual ferrite showed no increasing trend after holding at 600 °C but actually decreased. The decreasing trend was more pronounced under air-cooling conditions. This demonstrates that no α-ferrite precipitation occurs under actual production cooling rates. The cooling process promotes transformation of residual δ-ferrite into austenite. Moderately slow cooling below 600 °C facilitates further residual ferrite transformation.

## 4. Discussion

### 4.1. Morphology and Distribution Mechanism of Residual Ferrite in Continuous Casting Slab

Based on Figure 5, it can be observed that the primary morphologies of residual ferrite in the slab include long strip, skeletal, lathy and network structures. Ferrite morphology is typically influenced by the solidification mode, cooling rate and macrostructure [49,50,51,52,53,54]. In this study, the solidification mode across the entire section of the 304L slab was consistently the FA mode. The formation of long strip ferrite at the slab edges is attributed to the high cooling rate within the edge chill layer. This high cooling rate promotes nucleation of both ferrite and austenite phases but suppresses ferrite growth, preventing it from developing into a complete skeletal structure, hence resulting in the long strip ferrite morphology at the slab surface. In the columnar dendritic zone of the slab, the predominant ferrite morphologies are lathy and skeletal. Within this zone, the cooling rate decreases, providing sufficient kinetic conditions for ferrite growth. Consequently, secondary dendrites can develop from the long strip ferrite, forming densely interconnected skeletal ferrite. In the central equiaxed grain zone, the ferrite primarily exhibits a network morphology. Here, ferrite nucleates extensively within the liquid phase, growing with an equiaxed grain morphology. As ferrite continuously transforms into austenite, the austenite/ferrite interface moves toward the ferrite side. Ultimately, continuous network ferrite forms along the austenite grain boundaries.

Based on the three-dimensional map of residual ferrite content in the triangular zone of the continuous casting slab, the high cooling rate in the chill layer at the slab edge suppresses ferrite growth, resulting in lower ferrite content. However, at the 0 mm width position in the triangular zone, the ferrite distribution along the thickness direction exhibits a “V”-shaped profile, with higher levels on both sides and lower in the center. This occurs because solidification near the corner lags behind that at the center of the edge. The cooling rate at the corner is lower than at the center of the edge along the thickness direction. Consequently, at the 0 mm width position, ferrite on both sides (corners) has more time to grow, leading to higher ferrite content at the corners compared to the center. This creates the “V-shaped” ferrite distribution along the thickness direction. Between 20 mm and 120 mm in the slab width direction within the triangular zone, the cooling rate gradually decreases from the edge toward the center. This progressively enhances ferrite growth kinetics, causing ferrite content to increase steadily from the edge to the center, forming an “A”-shaped distribution. However, outside the triangular zone (width direction: 120~240 mm), the higher cooling rate at the edge inhibits the growth of high-temperature δ-ferrite, resulting in a lower ferrite content. As the distance from the surface toward the center increases, the cooling rate decreases. This leads to a gradual prolongation of the entire solidification and solid-state phase transformation process, while the ferrite dendrite arm spacing increases sharply. Concurrently, the extent of solid-state phase transformation weakens, causing a slight increase in room-temperature ferrite content. The decrease in ferrite content at the center is likely due to the sparse network-like ferrite structure formed there. The low cooling rate in the center allows more ferrite to transform into austenite, forming this sparse network-like ferrite structure, which ultimately reduces the ferrite content. Consequently, the ferrite content exhibits a distinct “M”-shaped distribution.

### 4.2. Mechanism of the Effect of Heat Treatment on Residual Ferrite Content and Distribution Throughout the Slab Thickness

During the heat treatment process throughout the entire thickness of the slab, the primary reaction occurring is the solid-state phase transformation of ferrite to austenite. This solid-state phase transformation is predominantly influenced by atomic diffusion distance. The heat treatment results show that the ferrite content decreased from the original 8.96% in the slab to varying levels, indicating that both heat treatment temperature and duration affect ferrite content. At 1100 °C, the temperature is relatively low, and the kinetic conditions for elemental diffusion are insufficient. Even with extended heat treatment time, the reduction in ferrite content remains limited. Thermodynamic calculations reveal that the temperature range of 1100–1236 °C corresponds to a single-phase austenite region. Holding within this range effectively promotes the transformation of ferrite to austenite. At 1250 °C, the thermodynamic results indicate that this temperature is no longer within the single-phase austenite region, and a small amount of ferrite phase may appear. However, due to its low content at this stage, ferrite remains metastable during heat treatment, while austenite persists as the primary stable phase. Additionally, the atomic diffusion rates at 1250 °C are higher than at 1200 °C. Consequently, the greatest reduction in ferrite content occurs under all tested conditions at 1250 °C. Specifically, under air cooling after holding at 1250 °C for 48 min—where atomic diffusion rates and diffusion time are maximized—the ferrite content decreases most significantly. At 1300 °C, thermodynamic calculations show ferrite content reaching as high as 20%. At this temperature, competing stability dynamics emerge between the two phases, with ferrite becoming more stable and less prone to transform into austenite. As a result, the net reduction in ferrite content is less substantial compared to that achieved at 1250 °C.

The heredity of the “M”-shaped distribution was also observed in the heat treatment results across the entire thickness of the slab. Under different holding times and cooling methods at 1250 °C, a distribution similar to the original slab’s “M”-shaped pattern appeared consistently. This heredity of the “M”-shaped distribution is primarily related to the initial ferrite content and secondary dendrite arm spacing of the slab. In the edge region along the slab thickness direction, the ferrite content remained similar across different holding times and cooling methods. This indicates that the transformation from ferrite to austenite was essentially complete under the conditions of 1250 °C, 32 min holding and water cooling. Further extending the holding time or reducing the cooling rate, while increasing the atomic diffusion distance, did not promote additional ferrite-to-austenite transformation. Consequently, only a short atomic diffusion distance is required to complete the solid-state phase transformation reaction at the slab edge. In contrast, within the central region (50–150 mm from the surface), the ferrite content decreased with prolonged holding time and reduced cooling rate. In this region (50–150 mm), the required diffusion distance for the solid-state phase transformation from ferrite to austenite increases. Here, extending the holding time and lowering the cooling rate effectively increases the atomic diffusion distance, thereby promoting the solid-state phase transformation. Under these conditions, the post-heat-treatment ferrite content mainly depends on the initial ferrite content.

The findings of this study provide theoretical guidance for steel manufacturers. Based on slab composition, manufacturers can first analyze the equilibrium solidification process using Thermo-Calc thermodynamic software. Below the δ-ferrite disappearance temperature during equilibrium solidification, maximizing heat treatment temperature and extending holding time effectively reduces residual ferrite content, whereby air cooling demonstrates superior efficacy over water cooling. Special attention should be given to the M-shaped ferrite distribution, particularly the bilateral ferrite peak zones along the slab centerline. Implementing dynamic intensive cooling in these critical regions suppresses ferrite coarsening.

## 5. Conclusions

1. This study investigated 304L austenitic stainless steel continuous casting slab. Along the thickness direction from surface to center, ferrite morphologies transition sequentially as long strip → skeletal/lathy → networked structures. In the width-center thickness direction, ferrite content varies within 3–13%, exhibiting an “M”-shaped distribution. Within the slab’s triangular zone, ferrite content ranges from 1.8% to 12.2%. The average residual ferrite along the thickness direction shows an “M”-shaped distribution, while along the width direction, the average ferrite content is lower at the edges (3.04%) and remains relatively consistent (8–10%) within the slab interior.
2. Thermodynamic software calculations of the slab’s equilibrium solidification process confirmed that the solidification mode of the 304L continuously cast slab follows the ferrite–austenite (FA) mode. Based on these thermodynamic calculations, heat treatment experiments were designed across the entire slab thickness. The results revealed that at a heat treatment temperature of 1250 °C, ferrite content decreased most significantly. Prolonged holding time and reduced cooling rate both facilitated further reduction in residual ferrite content. However, at 1300 °C (where the equilibrium microstructure consists of high-temperature δ-ferrite and austenite), the reduction in residual ferrite was less pronounced compared to the 1250 °C condition. Moreover, after heat treatment under various parameters, the ferrite distribution within the slab retained the original “M”-shaped profile observed in the as-cast slab.
3. Holding experiments at 600 °C confirmed no α-ferrite precipitation under actual cooling conditions. Moderately slow cooling below this temperature further promotes the transformation of high-temperature δ-ferrite into austenite.

## Figures and Tables

**Table 1:** Main chemical components of 304L slab.

C	Si	Mn	P	S	Cr	Ni	N
0.0154	0.3763	1.795	0.0318	0.0014	18.01	8.01	0.0696

**Table 2:** Heat treatment test scheme for 304L continuous casting slab.

	Holding Time	1000 °C	1200 °C	1250 °C	1300 °C
Water cooling	32 min	1	5	9	13
	48 min	2	6	10	14
Air cooling	32 min	3	7	11	15
	48 min	4	8	12	16

**Table 3:** Distribution table of residual ferrite in 304L continuous casting slab.

Thickness Width	10	20	40	60	80	100	120	140	160	180	200	220	240
10	4.8	5.2	3.7	2.7	2.4	2.6	2.8	2.2	2.6	2.0	3.1	2.6	2.6
20	5.4	7.1	9.0	10.7	9.6	8.9	8.2	8.0	7.0	5.3	6.7	7.0	7.5
40	2.5	8.7	6.9	9.8	8.9	9.8	10.3	10.2	9.5	8.7	9.0	8.8	10.8
60	2.0	9.7	10.4	9.8	11.1	11.1	11.6	10.8	10.6	9.9	10.9	9.2	11.8
80	1.9	10.0	10.8	11.0	10.6	10.4	10.7	12.5	11.8	12.4	11.2	11.3	12.4
100	1.8	9.8	11.1	10.8	10.3	11.5	12.2	9.6	9.1	9.5	8.3	10.9	11.2
120	1.8	9.1	9.7	11.2	11.3	11.0	11.2	12.4	11.6	11.7	12.6	12.2	12.3
140	2.0	9.9	10.2	9.7	11.4	11.6	11.6	11.7	11.5	10.9	10.7	11.7	10.7
160	2.3	8.9	9.2	10.2	10.2	9.9	9.8	10.7	10.2	9.4	10.1	8.7	10.0
180	3.9	7.4	8.0	8.6	9.9	8.7	8.0	9.2	8.9	8.1	6.6	8.8	8.4
200	5.0	4.0	3.7	3.2	3.3	2.4	2.7	2.2	2.7	3.2	2.3	2.6	3.2

## Data Availability

The original contributions presented in this study are included in the article. Further inquiries can be directed to the corresponding authors.
